# The effect of collaborative innovation on ICT-based technological convergence: A patent-based analysis

**DOI:** 10.1371/journal.pone.0228616

**Published:** 2020-02-04

**Authors:** Inyoung Hwang

**Affiliations:** Korea Institute of Science & Technology Evaluation and Planning, Chungcheongbuk-do, South Korea; Shandong University of Science and Technology, CHINA

## Abstract

Collaborative innovation is widely recognized as an instrument to promote technological convergence. However, its effects on technological convergence remain debatable. Using firm-level panel data of patenting in the Korean ICT industry from 1980 to 2015, I examine the effects of four collaborative innovation types (i.e., Inter-firm, Inter-ICT firm, Firm-University, and Firm-Government Research Institution (GRI) on ICT-based technological convergence. The results reveal the magnitude of Inter-ICT firm collaborative innovation was found to be significant and largest. The effects of the remaining three collaborative innovation types were significant but inconsiderable. Governments may consider the differential effects of collaboration types when designing incentive systems to promote technological convergence.

## Introduction

Technological convergence has been suggested as a means to (a) create new markets through the synergistic combination of different technologies and (b) provide technical and economic benefits for the industry as a whole [1–4]. It can positively influence societal productivity as well as the nation’s economic growth [5]. Recent ICT-based technological convergence shows that such an expectation is about to be met. For instance, intelligent vehicles are a prime example of technological convergence that combines mechanical and ICT technologies. While not always the case, technological convergence often results from the collaborative innovation of two or more entities [6].

Several countries have implemented policies to facilitate technological convergence by supporting collaborative innovations. The U.S. government has supported R&D infrastructure, a collaboration network between firms, and human resource development [7]. European countries have pursued convergence strategies within the EU Framework Program (FP) [2], which includes strengthening multilateral cooperation, identifying creative ideas, enhancing researcher liquidity, and strengthening European collaborative research capabilities [7]. East Asian countries such as Korea and Japan have also implemented government-led technological convergence policies focused on activating collaborative innovation. The investment in collaborative innovation to promote technological convergence has become a popular policy instrument worldwide.

However, there is limited empirical evidence that collaborative innovation promotes technological convergence. There are different types of collaborative innovations discussed in the literature, including firm–university, firm–government, research institution (GRI), and inter-firm collaborative innovation. Different types of collaborative innovation may not affect technological convergence in the same manner or magnitude. Thus, allocating resources differently for different types of collaboration may be efficient. The purpose of incentive systems is to facilitate a collaborative innovation ecosystem in the long term, so it is necessary to identify whether and which collaborative innovation types promote technological convergence most frequently.

In this paper, I examine whether firms’ collaborative innovation promotes technological convergence and which type of collaborative innovation promotes technological convergence best in the context of the Korean ICT industry. Korea has had one of the most developed ICT infrastructures in the world due to its intensive investment in the ICT industry since the end of the 1990s and has always maintained the highest level of international ICT development indicators [8]. The Korean ICT industry also has a high level of international technology and R&D investment, including a large number of global firms in semiconductors and telecommunication. It provides a fertile ground in which to investigate the effect of different types of collaborative innovations on ICT-based technological convergence.

The remainder of the paper is organized as follows. Section 2 reviews the literature on the relationship between collaborative innovation and technological convergence and develops the research hypotheses. Section 3 presents the data and methods, Section 4 presents the analysis results, and Section 5 offers a discussion of the findings.

## Literature review

Technological convergence refers to a combination of different technologies used to solve a technological problem [9]. Rosenberg [10] coined the term, technological convergence, defined as a communal technological innovation that takes place in the process of various industries resolving their technical problems. Kodama [11] presented the term technology fusion, which refers to the transformation of core technologies. Pennings and Puranam [12] proposed differentiating technological convergence from technology fusion. They argued that technology fusion is simply a combination of existing technologies, but that technological convergence is a combination of existing technologies that also aims to create innovations no previously in existence. Technological convergence, combined with market convergence, ultimately leads to industry convergence [13]. According to this view, technological convergence arises from the transfer of scientific achievements to industry, obscuring the boundaries between industries with product or service innovation. Therefore, technological convergence is considered to arise from the transfer of accumulated knowledge to the industry as science advances.

Previous research on technology convergence has focused primarily on classification and prediction based on technology interdependencies at industry or technology level [14–18]. However, the importance of firm-level analysis has also been emphasized in recent literature that it can provide useful implications for the firm’s strategic decision-making to survive in R&D competition [19]. Firms can seek for collaborative innovation with other firms that have different technologies to reduce the time required for innovation and to respond to the market demand [20–23]. Given recent technological developments and the rapid progress of convergence among various technologies, it is increasingly difficult for firms to meet multiple demands in the market through internal innovation; thus, the importance of collaborative innovation is increasing. Technological convergence allows firms to use a variety of strategies to implement innovations in an environment in which the technology boundaries are ambiguous [24]. Firms prefer internal innovation for their core competencies, but, as uncertainties in the market environment increase, they also utilize resources from outside the firm [25]. According to resource-based theory, firms complement the assets and capabilities needed for innovation through collaboration [21–23, 26, 27] and reduce innovation costs [23, 28]. Firms can overcome the limitations of R&D costs and the shortening of product life cycles by cooperating with another firm rather than internal firm innovation [29].

ICT-based technological convergence has an advantage in that it has a greater impact than other forms of technology-based convergence; it is also easier to commercialize and, thus, stimulates economic growth [18, 30, 31]. It has the most diverse convergence characteristics when compared to other technology-based convergence, involving the convergence of products, services, and industries [31]. ICT is regarded as a general purpose technology (GTP), which fuses with all industries for an extended period of time, thereby making the production process more efficient; GTPs also commonly promote innovation in other industries. ICT-based technological convergence creates new products and services that did not exist previously, changes existing market structures, creates new markets, and promotes innovation in other technology areas [32]. Due to these advantages, countries including the United States, Japan, Korea, and countries in the EU are implementing policies to promote ICT-based technological convergence.

The ICT industry is one of the most innovative industries, characterized by high R&D intensity and a rapid innovation cycle. Given the characteristics of the ICT industry, ICT firms are sensitive to changes in technology demand in the market and are particularly sensitive to changes in demand for convergent technology. With the high sensitivity and high degree of R&D intensity, ICT firms may have a greater incentive to develop convergent technology than firms in other industries. The collaborative innovation activities of ICT firms can have a positive impact on technological convergence. In other words, ICT firms can (a) produce convergent technology by strategically cooperating with other firms to meet the demand for convergent technology in the market and (b) gain an advantage in the innovation competition.

Although previous studies have reported some mixed results, they imply that collaborative innovation between innovation entities in the ICT industry may increase the probability of combining heterogeneous technologies, which can lead to technological convergence. An earlier study by Duysters and Hagedoorn [6] addressed that the relationship between firm alliances and IT-based technological convergence is unclear. Lee, Lee, Song, Kim [33] revealed that inter-firm collaborations involving large firms and GRIs promote ICT-based technological convergence. However, Lee and colleagues [33] do not consider a time variable, they could not control for the possibility of bias resulting from cross-sectional analysis in innovative research (cf., [34]). These studies focused primarily on adding statistical data to conceptual discussions [35], performing descriptive analyses based on the collected data [33], or analyzing differences between collaborative and non-collaborative studies [36]. Thus, there is a limitation to infer the effects of collaborative innovation on ICT-based technological convergence from these results.

In addition, motivations can differ, depending the type of partner participating in collaboration [37, 38]. For instance, firms with strategies for minimizing conflicts related to intellectual property tend to choose universities as cooperating partners [39]. Firms choose different types of collaboration partners, such as universities, GRIs, or firms, depending on their different strategies, and patterns of innovation can vary according to the collaboration type [38, 40, 41]. The question of which type of collaborative innovation has the greatest effect on technological convergence can be raised.

The major innovators in the ICT industry—GRI, university, and firm—have different organizational characteristics, which can affect innovation performance. According to the component model of creativity, organizational autonomy is one of the most important factor influencing innovation performance [42–44]. The GRI has been regarded as having relatively little autonomy when compared to other types of organizations because it relies on most of its budget for government contributions [45]. The Korean Government has supported innovation facilities, workforce, and knowledge through the GRI for firms that do not have their innovation capabilities. Given that the GRI has mainly been used as a policy instrument to supplement firms’ lack of innovation capabilities, firm–GRI collaborative innovations can be seen as a result of the incentive to reduce the innovation costs of firms with insufficient innovation capacity rather than the convergence of heterogeneous technologies to meet the diversified technology demand of the market.

Universities also playing the role of lending the resources needed for innovation to the firms lacking innovation capacity in the National Innovation Systems (NIS) [38, 39, 46]. While the government directly controls GRI innovation, universities have greater autonomy than GRIs in selecting innovation subjects because they are indirectly controlled through incentives such as research funding. Although universities are guaranteed greater autonomy than the GRI, it is difficult to say that they are as sensitive to changes in the technology demand in the market as firms.

High-technology firms such as ICT firms are more exposed to the risks from technological change than non-innovative firms [47]. Thus, firms can seek collaborative innovation with other firms that have different technologies to reduce the time required for innovation and to respond to market demand [20]. In innovation-intensive industries like the ICT industry, innovation relates to the firm’s growth, so having an advantage in innovation competitiveness is also important to the survival of the firm. Therefore, this study assumes that the frequency of collaborative innovation as a response to technology needs in the market may be higher in inter-firm collaborative innovation than in other types of collaborative innovation.

## Data and methods

### Data

This study constructs firm-level panel dataset using patent and firm data. I collected patent data from Korean ICT firms and constructed an unbalanced panel dataset of years ranging from 1980 to 2015. Korean ICT firms are defined by KIS-Industry Classification (KIS-IC). KIS-IC is an industry classification standard based on the Global Industry Classification Standard (GICS) jointly developed by S&P and MSC. It categorizes all Korean industries into ten sectors, 23 industry groups, 29 industries, and 122 sub-industries. A total of 525 Korean firms were classified as ICT firms by KIS-IC at the time of analysis. The panel data constructed in this study include all 525 Korean ICT firms. However, given that the ICT firms defined by KIS-IC may change over time, the sample can be considered as a list of available firms among all Korean ICT firms.

Patents are those registered only in Korea. This restriction is to prevent the possibility of overestimation due to the ability to register the same patent in multiple countries. Patent data are collected from the National Digital Science Library (NDSL). NDSL is a national science and technology database operated by the Korea Institute of Science and Technology Information (KISTI) under the Ministry of Science and ICT (MSIT). It includes all patent information filed and registered with the Korean Intellectual Property Office (KIPO). To collect all the patent data filed by Korean ICT firms during the 36 years, I collected data by specifying 525 firm names in the applicant portion of the patent search formula. In the case of a firm that changed its name, I also include the previous name. I merged the patent and firm data and then converted these data into firm-level panel data based on the applicant and filing year. The dataset includes 230,388 patent data cases during the period. The dataset also includes patent name, patent number, filing date, registration date, publication date, applicant information, and International Patent Classification (IPC) code.

After assigning a unique id to each firm, I construct the firm data by adding the year data to each patent using the year information in the patent application number. These data are then matched with firm data. When matching data, I also include firms that never filed a patent in the dataset to eliminate the possibility of selection bias. The panel data include a total of 9,882 firm-level observations over the same period mentioned above. The firm data are collected from the KIS-Value software, which allows access to the firm-level database. KIS-Value is a firm database operated by NICE Information Service that provides disclosure data including financial information of about 20,000 Korean firms in the form of panel datasets. It contains firmcharacteristic data in all Korean industries defined by KIS-IC, including the ICT industry. However, since the R&D expenditure data collected by KIS-Value have many missing values, additional R&D expenditure data are collected from the annual business reports published by the Data Analysis, Retrieval and Transfer system (DART), an electronic disclosure system of the Financial Supervisory Service (FSS) under the Financial Services Commission (FSC). It contains all disclosures, including business reports submitted directly by Korean firms. The business report provides detailed disclosure information such as sales, number of employees, assets and liabilities. This study supplemented the missing values of the data collected by KIS-Value by collecting firm data specified in the business report. DART provides business reports from 1999 to the present. In sum, there are 525 firms over 35 years, including those firms created or listed between 1980 and 2015. Therefore, this dataset contains only the initially selected 525 ICT firms in the unit of analysis and does not include non-ICT firms. Non-ICT firms appear in “Collaborative innovation with non-ICT firms” among the types of joint patents generated by ICT firms.

### Variables

#### Dependent variable

Technological convergence is measured as the number of convergent patents registered by ICT firms based on co-classification using the patent’s IPC code (Curran & Leker, 2011). The IPC code of each patent by sector is classified according to the IPC-Technology Concordance Table in World Intellectual Property Indicators 2016 [48]. The table categorizes technologies at the sub-class level and includes five technology sectors and 35 technology fields. Heterogeneous technologies are defined in a more structured form than the Section-Class-Sub-class classification, where some technology fields include only a single sub-class, while some fields include various sections. In this study, if the IPC codes of a patent belonging to different technological sectors (e.g., electrical engineering and chemistry), the patent is considered a convergent patent.

In order to measure technological convergence, this study assigned one or more technology sectors to each patent based on the IPC information included in each patent from the raw patent data. Through this, I identified whether each patent is a convergent patent. The unit of analysis was then converted from patent to applicant to measure the number of convergent patents filed by a specific applicant in a specific year.

#### Independent variables

Collaborative innovation was measured by the number of joint patent activities registered by ICT firms [49–52]. The ratio variable, as the number of collaborative patents to total patents, was not used in this study because there is a risk of overestimating the preference of collaboration among firms with a low frequency of innovation. In an industry with a high percentage of firms with a low frequency of innovation, such as the Korean ICT industry, biases due to this overestimation can be significant. Thus, this study measures collaborative innovation with a count variable instead of a ratio variable. Each joint patent was restricted to a patent filed by two or more legal entities such as a firm or university. Patents co-filed with both firms and individuals are not regarded as joint patents because most of the individuals are employees or representatives of the firm.

Joint patenting, the measure for collaborative innovation, in this study includes five types: *Collab*.*Innov*., *Firm-University*, *Firm-GRI*, *Inter-firm*, and *Inter-ICT firm*. *Collab*.*Innov*. refers to joint patents regardless of collaboration types, *Firm-University* for joint patents registered by firm-university collaboration, *Firm-GRI* for joint patents registered by firm-GRI collaboration, *Inter-firm* for joint patents registered by inter-firm collaboration regardless of industries, and *Inter-ICT Firm* for joint patents registered by inter-ICT firm collaboration. The patent data include 16 joint patents filed by more than two entities such as Firm-University-GRI collaboration. *Inter-firm* includes patents filed by ICT firms in collaboration with ICT firms as well as non-ICT firms.

This study classified applicants based on applicant information included in each patent from the raw patent data to measure independent variables. Specifically, this study identified whether each patent is a collaborative patent and what type of collaboration it went through. The unit of analysis was then converted from patent to applicant to measure the number of various types of patents filed by a particular applicant in a particular year. The number of joint patents for each collaboration type is shown in Table 1.

**Table 1:** Descriptive statistics of joint patents(1980–2015).

Type of Collaboration	Observations
Joint Patents (Total)	9,143
** Number of Collaborators**	
Joint Patents (2 entities)	8,796
Joint Patents (3 entities)	75
Joint Patents (4 entities)	109
Joint Patents (5 entities)	163
Total	9,143
** Type of Collaborators**	
Inter-firm	2,416
Firm—University	2,192
Firm—GRI	4,330
Firm–University—GRI	16
Firm–University (Foreign)	189
Total	9,143

Korean ICT firms have performed more than 9,000 collaborative innovations over 35 years. All collaborators can be classified into firms, universities, GRI, and foreign universities. The scope of this study is a collaborative innovation in the Korean ICT industry, so foreign universities are excluded from the analysis. Trilateral collaborations involving all of the firm, university, and GRI were carried out 16 times, indicating that it has been rarely performed. This study excludes trilateral cooperation in the analysis because it has few observations and is not only suitable for regression analysis but also has insufficient significance.

#### Control variables

Since the Schumpeterian hypothesis on the relationship between firm size and innovation, many researchers have argued that firm size affects patent performance. I control for firm size, measured by the logarithm of the number of workers as previous studies [38, 53].

Innovation studies have suggested that R&D expenditure has a positive effect on a firm’s patent performance [54]. Griliches [34] and Crepon et al. [55] also report that R&D investment is a major factor in patent performance. I use the R&D expenditure as a control variable that affects the patent performance, and I convert the annual R&D expenditure of each firm into the unit of 1 million KRW by log scale [56].

Recent studies of innovation factors revealed that firms’ productivity, represented by capital intensity, affects patent performance. Hall and Ziedonis [53] empirically demonstrated the effect of capital intensity on patent applications of semiconductor firms. Bessen and Hunt [57] found that the magnitude and direction of the effect of capital intensity on a firm’s patent performance, similar to the findings by Hall and Ziedonis [53]. Czarnitzki, Kraft, and Thorwarth [58] determined that capital-intensive firms rely more heavily on technology, and that they tend to retain their intellectual property rights through patent applications. I control for firm productivity, measured by the annual capital intensity of each firm (converted into the unit of 1 million KRW by log scale). Hong and Su [59] found that there is a positive correlation between firm age and collaborative innovation. Initial Public Offering (IPO) status should be controlled in this study because it is an endogenous variable that affects innovation. Bernstein [60] found that IPO is an endogenous variable that affects innovation. I also use conglomerate affiliates and large firms as control variables to control for the outliers that produce the majority of patents such as Samsung and LG. Lastly, I control for time effect by using the year as a control variable.

### Models

I used a negative binomial model because the dependent variable is a count data point. Estimates are inconsistent when estimating data with over-dispersion by the Poisson model [61]. The over-dispersion test of technological convergence shows that the Variance-to-Mean Ratio (VMR) is quite high at about 120.26, suggesting that it is appropriate to perform regression analysis with the panel negative binomial model. The specification of the panel negative binomial model is as follows:

$${TechConv}_{it}=\alpha +\beta_{1}X_{it}+\beta_{2}Ln{\left(FirmSize\right)}_{it}+\beta_{3}Ln{\left(R\&D\_Exp.\right)}_{it}+\;\beta_{4}Ln{\left(Productivity\right)}_{it}+\;\beta_{5}{Conglomerate}_{it}+\;\beta_{6}FirmAge_{it}+\;\beta_{7}LargeFirms_{it}+\;\beta_{8}IPO_{it}+\;\beta_{9}Year+\delta_{i}+\varepsilon_{it}$$ (1)

(*X*_*it*_ = *Collab*.*Innov*., *Firm-University*, *Firm-GRI*, *Inter-firm*, *Inter-ICT firm*).

I estimated the fixed effect model and the random effect model, respectively, and then determined which model was more appropriate by using the Hausman test.

The potential problem of endogeneity must be considered to estimate causality. Since the firm is the subject of decision making, the same firm decides both collaborative innovation and technological convergence. Additionally, collaborative innovation affects technological convergence, but technological convergence can also affect collaborative innovation. In this case, it is necessary to estimate using an instrumental variable (IV). Yamamura [62] notes that when analyzing count data with over-dispersion, the IV negative binomial model has not yet been developed and the IV Poisson model should be used. I use the IV Poisson GMM model proposed by Mullahy [63]. The lagged explanatory variable can be used as an instrument since it is correlated with the original independent variable, and also correlated with the error term but not contemporaneously correlated because it is lagged [64–66]. Therefore, I use lagged value of each independent variable as an instrumental variable. Balsmeier, Buchwald, and Stiebale [67] suggest that an additional test can be performed on the linear 2SLS model to ensure the robustness of the IV estimator when estimating the IV Poisson GMM model. I performed a weak IV test on the linear 2SLS model for the robustness check on the IV estimator. The specification of IV Poisson GMM model for endogenous variable *X*_*it*_ is as follows:

$${TechConv}_{it}=exp(\alpha +\beta_{1}X_{it}+\sum_{n=1}^{c}{{\beta_{n}X}^{n}}_{it})+\varepsilon_{it}$$ (2)

Where *X* stands for the independent variable, *X*^*n*^ stands for control variables, and *c* stands for the number of control variables. The moment condition in which *X*_*it*−1_ presents an instrumental variable is:

$$E[{TechConv}_{it}-\;exp(\alpha +\beta_{1}X_{it}+\sum_{n=2}^{8}{{\beta_{n}C}^{n}}_{it}+\;\beta_{9}Year)|X_{it},\;X_{it-1}\;]=0$$ (3)

I check whether there is an endogeneity problem due to simultaneity in the independent variables using the Hausman test on the Poisson and IV Poisson GMM estimates. If there is an endogeneity problem, the IV Poisson GMM estimation result is adopted. Otherwise, the panel negative binomial estimation result is adopted.

## Results

### Descriptive analysis

The descriptive statistics for the completed dataset appear in Table 2. The means and standard deviations of *technological convergence* and *Collab*.*Innov*. indicate that a small number of firms led the innovation of the Korean ICT industry. The standard deviation of *technological convergence* is ten times greater than the average, indicating that some market-dominant firms are producing most of the technological convergence in the industry. The standard deviation of the independent variables is also more than ten times greater than the mean, indicating that collaborative innovation is concentrated in a small number of firms. The mean of *Collab*.*Innov*. is close to 1, and most firms seem to experience collaborative innovation; however, the standard deviation is greater than 15, indicating that collaborative innovation is concentrated in a small number of firms. The mean of collaborative innovation by type is in the order of *Firm-GRI*, *Inter-firm*, *Firm-University*, and *Inter-ICT firm*. From the dummy variables, it is apparent that 78% of the firms surveyed are conglomerate affiliates, 37% are large firms, and 47% are listed firms. The average age of all firms is 12 years.

**Table 2:** Descriptive statistics of the variables (1980–2015).

Variable	Obs.	Mean	Std. Dev.	Min.	Max.
**Dependent Variable**					
Technological Convergence	9,882	0.8279	9.9783	0	431
**Independent Variable**					
Collab.Innov.	9,882	1.0041	15.2074	0	579
Firm-University	9,882	0.2088	4.9326	0	287
Firm-GRI	9,882	0.5074	11.3525	0	481
Inter-firm	9,882	0.2727	2.7625	0	125
Inter-ICT firm	9,882	0.0578	0.7375	0	32
**Control Variable**					
Ln(Firm Size)	8,661	4.9529	1.3908	0	11.5324
Ln(R&D Exp.)(mil.)	7,090	7.2874	1.8118	-1.9290	16.5450
Ln(Productivity)(mil.)	8,661	5.4180	1.0995	-0.3352	10.7682
Conglomerate Affiliates	9,882	0.7816	0.4131	0	1
Firm Age	9,882	12.1258	10.0161	0	66
Large Firms	9,882	0.3760	0.4844	0	1
IPO	9,882	0.4744	0.4994	0	1
Year	9,882	2004.237	8.0585	1980	2015

**Fig 1:**
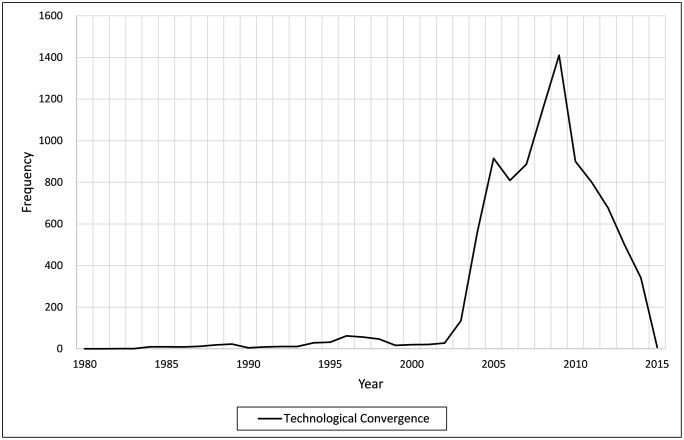
Technological convergence by year.

Fig 1 presents the number of technological convergences by year since 1980. *Technological convergence* increased slightly at the end of the 1990s, but decreased steadily until the early 2000s. Since 2003, *technological convergence* increased significantly, peaking in 2009 and then declining, reaching a shallow level in 2015. This trajectory relates to the steady decline in the number of patents filed by Korean ICT firms, after peaking in 2005.

**Fig 2:**
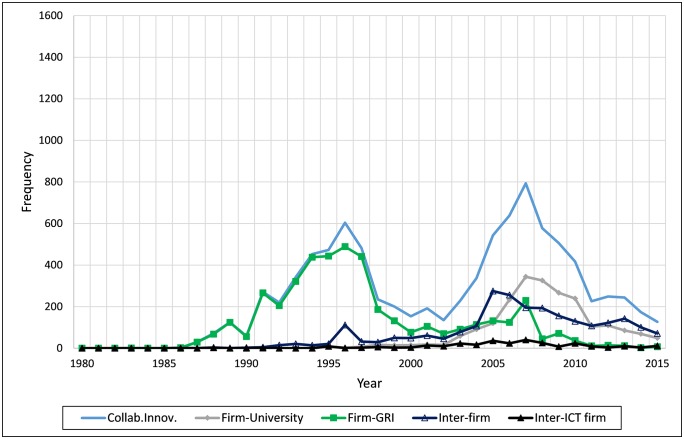
Collaborative innovation by year.

Fig 2 presents changes in collaborative innovation by year. *Firm-GRI* was high until the 1990s, and most of the collaborative innovations undertaken by ICT firms were part of a government-led initiative. In the 2000s, the gap between *Collab*.*Innov*. and *Firm-GRI* increased. In particular, since the mid-2000s, the proportion of *Inter-firm* and *Firm-University* has increased. Moreover, since 2010, the proportion of *Inter-firm* has been the highest among all collaboration types. This finding indicates that the government-led collaborative innovation initiated by the end of the 1980s was a major part of the collaborative innovation by the end of the 1990s; it has gradually replaced by collaborative innovation between firms.

### Model estimations

I report the fixed effect model of panel negative binomial regression (FENB) to show the effect of collaborative innovation on technological convergence based on the Hausman test result (see S2 Table for the Hausman test result). I separately report the random effect model (RENB) in S1 Table. The estimation results of the FENB model appear in Table 3.

**Table 3:** FENB regression.

Variable	Model (1)	Model (2)	Model (3)	Model (4)	Model (5) ^a^
**Independent Variables**					
Collab.Innov.	0.0039*** (0.0005)				
Firm-University		0.0072*** (0.0011)			
Firm-GRI			0.0049*** (0.0010)		
Inter-firm				0.0226*** (0.0025)	
Inter-ICT firm					0.0603*** (0.0100)
**Control Variables**					
Ln(FirmSize)	0.0848 (0.0586)	0.1263* (0.0589)	0.0756 (0.0599)	0.1150* (0.0581)	0.0988 (0.0571)
Ln(R&D Exp.)	0.1135** (0.0433)	0.0980* (0.0439)	0.1176** (0.0438)	0.0966* (0.0431)	0.1058* (0.0431)
Ln(Productivity)	-0.0214 (0.0838)	0.0060 (0.0835)	-0.0268 (0.0840)	-0.0327 (0.0835)	-0.0300 (0.0823)
Conglomerate Affiliates	-0.5393* (0.2531)	-0.5436* (0.2528)	-0.5380* (0.2529)	-0.5490* (0.2536)	-0.5653* (0.2322)
Firm Age	-0.0132 (0.0072)	-0.0150* (0.0072)	-0.0126 (0.0072)	-0.0170* (0.0072)	-0.0155* (0.0071)
Large Firms	-0.0507 (0.1918)	-0.0804 (0.1910)	-0.0480 (0.1914)	-0.0677 (0.1916)	(Omitted)
IPO	-0.4243*** (0.1277)	-0.3908** (0.1275)	-0.4364*** (0.1291)	-0.5127*** (0.1291)	-0.4223*** (0.1269)
Year	0.1294*** (0.0106)	0.1266*** (0.0105)	0.1298*** (0.0105)	0.1302*** (0.0105)	0.1299*** (0.0101)
**Constant**	-261.2291*** (21.0624)	-255.7600*** (20.9549)	-262.0004*** (20.9034)	-262.7715*** (20.9869)	-262.1929*** (20.1728)
**Observations**	3,849	3849	3849	3849	3849
**Log Likelihood**	-2635.7803	-2641.7682	-2643.8136	-2632.6903	-2641.7891
**LR Chi-squared**	834.02***	822.04***	817.95***	840.20***	822.00***
**Prob > Chi-squared**	0.0000	0.0000	0.0000	0.0000	0.0000

Note:

* p<0.05;

** p<0.01;

*** p<0.001;

Standard errors in parentheses.

^a^ In the case of Model (5), the log-likelihood estimation failed until the maximum iteration in the model estimation. Instead, the model is estimated excluding the *Large Firms* variable.

Five regression models are estimated to compare the effect of various types of collaborative innovation on technological convergence. I examined the correlation between the variables before performing regression analysis to detect multicollinearity. The results show that most of the variables have moderate or weak correlations with each other. The Pearson correlation coefficient between the year and Ln (Productivity) is the highest at 0.6384, so it is reasonable to assume that there is no risk of multicollinearity in this model. Overall, collaborative innovation (*Collab*.*Innov*.) has a positive effect on technological convergence. Four collaborative innovation types also have statistically significant positive effects in all models. The magnitude of the effect is the greatest for the *Inter-ICT firm* variable, followed by *Inter-firm*, *Firm-University*, *Firm-GRI*, and *Collab*.*Innov*. That is, inter-ICT firm collaborative innovation has a larger effect on technological convergence than other types of collaborative innovation.

Next, IV Poisson GMM estimation was performed with the robust standard error. Stock and Yogo [68] suggested that, when the F-statistic of the first-stage regression is less than 10, the corresponding instrumental variables are weak and the estimates are seriously biased. In all models used in this study, the null hypothesis that the instrumental variables are weak is rejected. The estimation of the IV Poisson GMM model appears in Table 4.

**Table 4:** IV Poisson GMM with VCE(Robust).

Variable	Model (1)	Model (2)	Model (3)	Model (4)^a^	Model (5)
**Independent Variables**					
Collab.Innov.	0.0015 (0.0008)				
Firm-University		0.0035* (0.0014)			
Firm-GRI			-0.0012 (0.0026)		
Inter-firm				0.0214*** (0.0053)	
Inter-ICT firm					0.0697** (0.0258)
**Control Variables**					
Ln(FirmSize)	0.4564*** (0.1167)	0.4780*** (0.1093)	0.4928*** (0.1260)	(Omitted)	0.4713*** (0.1112)
Ln(R&D Exp.)	0.4193*** (0.0552)	0.4010*** (0.0553)	0.4349*** (0.0654)	0.5905*** (0.0401)	0.3390*** (0.0630)
Ln(Productivity)	-0.2569 (0.1739)	-0.2362 (0.1711)	-0.2467 (0.1814)	-0.4093** (0.1526)	-0.1806 (0.1690)
Conglomerate Affiliates	0.0428 (0.1422)	0.0432 (0.1413)	0.0266 (0.1420)	0.1448 (0.1340)	0.0502 (0.1406)
Firm Age	-0.0299* (0.0125)	-0.0317* (0.0124)	-0.0313* (0.0130)	(Omitted)	-0.0343* (0.0137)
Large Firms	0.5498*** (0.1429)	0.5552*** (0.1387)	0.4631** (0.1482)	0.9089*** (0.1211)	0.6661*** (0.1502)
IPO	-1.0111*** (0.2405)	-0.9055*** (0.2336)	-0.8666*** (0.2258)	-0.9001*** (0.1931)	-0.8569*** (0.2417)
Year	0.0728*** (0.0128)	0.0711*** (0.0125)	0.0666*** (0.0131)	0.0513** (0.0176)	0.0801*** (0.0139)
**Constant**	-151.0886*** (25.2727)	-147.6601*** (24.6526)	-138.9578*** (25.7383)	-106.5242** (34.6730)	-165.6213*** (27.5088)
**Observations**	6,851	6,851	6,851	6,851	6,851
**Weak IV Test**	152.18	372.36	77.69	264.40	233.65

Note:

* p<0.05;

** p<0.01;

*** p<0.001;

Standard errors in parentheses.

^a^ Model (4) cannot be estimated because the Hessian is not positive semidefinite. Therefore, it is estimated excluding the *Ln(FirmSize)* and *Firm Age* variables.

The results in Table 4 differ from the results of the FENB models in Table 3. Collaborative innovations have statistically significant positive effects in only three models. *Inter-ICT firm*, *Inter-firm*, and *Firm-University* affect technological convergence, but the remaining types of collaborative innovation do not affect technological convergence.

Hausman test between the IV Poisson GMM model and the Poisson model (see S3 Table) is performed to identify the presence of endogeneity due to simultaneity in the independent variables. The test results (see S4 Table) show that the estimated Chi-squared value is statistically significant only in Model (5), indicating that the IV Poisson GMM model is more suitable than the Poisson model only in Model (5). This result implies that endogeneity exists in the *Inter-ICT Firm* variable. The existence of endogeneity in the collaborative innovation variables means that there is simultaneity whereby technological convergence also affects collaborative innovation. This finding implies that firms use collaborative innovation strategically as a means to produce technological convergence. Therefore, the endogeneity test result can be interpreted that ICT firms utilize *inter-ICT firm* collaborative innovation among various collaborative innovation types as a strategic tool for technological convergence. This finding is consistent with the expectation that firms in the future IT industry will collaborate to promote technological convergence in response to the demand for new technology regimes [6].

### Incident rate ratio (IRR) and the interpretation of the models

To calculate the incident rate ratio (IRR), I use the estimation results of the FENB for models (1)–(4) and that of the IV Poisson GMM model for Model (5), which addresses endogeneity in the independent variable. Table 5 summarizes the estimated coefficients.

**Table 5:** Summary of the results.

	Model (1)	Model (2)	Model (3)	Model (4)	Model (5)
	FENB	FENB	FENB	FENB	IV Poisson GMM
Collab.Innov.	0.0039***				
Firm-University		0.0072***			
Firm-GRI			0.0049***		
Inter-firm				0.0226***	
Inter-ICT firm					0.0697**

Note:

* p<0.05;

** p<0.01;

*** p<0.001

The coefficients in Table 5 represent the change in the expected log value of technical convergence when the independent variable changes by one unit, and is defined as $\beta =log(\frac{\gamma_{x+1}}{\gamma_{x}})$. The coefficients can be interpreted through transformation into IRR, which is defined as $\frac{\gamma_{x+1}}{\gamma_{x}}$ and can be calculated through the transformation of $\frac{\gamma_{x+1}}{\gamma_{x}}=e^{\beta }$. In Table 6, IRRs show the effect of each type of collaborative innovation on technological convergence. The size of the estimated IRR is the largest for the *Inter-ICT firm*, followed by *Inter-firm*, *Firm-University*, *Firm-GRI*, and *Collab*.*Innov*. Again, *inter-ICT firm* collaboration has the largest effect on technological convergence among all the collaborative innovation types.

**Table 6:** IRR of technological convergence by various types of collaborative innovation.

	Model (1)	Model (2)	Model (3)	Model (4)	Model (5)
	FENB	FENB	FENB	FENB	IV Poisson GMM
Collab.Innov.	1.0039***				
Firm-University		1.0072***			
Firm-GRI			1.0049***		
Inter-firm				1.0228***	
Inter-ICT firm					1.0721**

Note:

* p<0.05;

** p<0.01;

*** p<0.001

**Fig 3:**
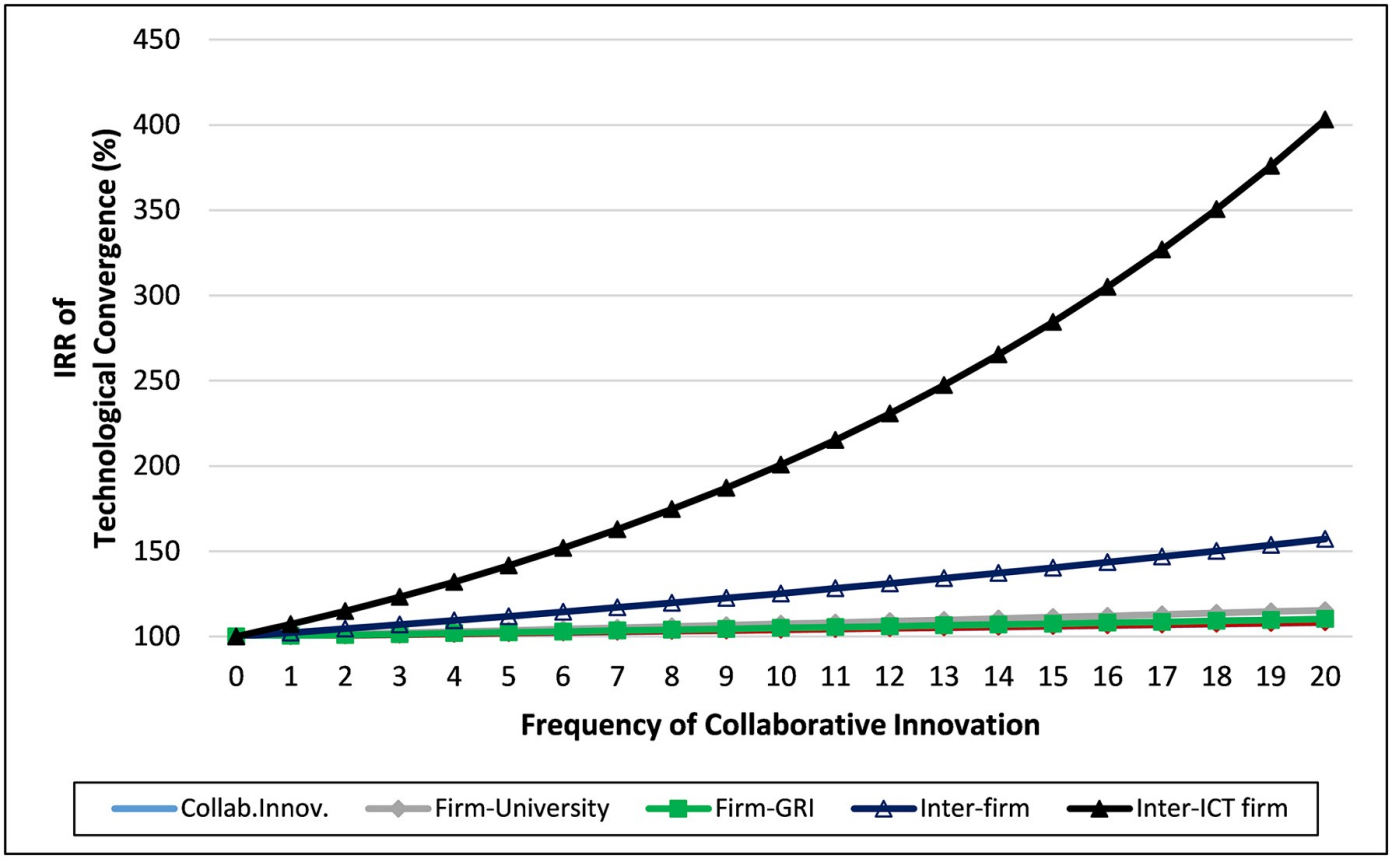
IRR of technological convergence by the frequency of collaborative innovation.

Fig 3 presents the expected IRR of technological convergence as collaborative innovation increases. As inter-ICT firm collaborative innovation increases by 10, technological convergence doubles; when it increases by 20, technological convergence increases by more than four times. Inter-firm collaborative innovation including non-ICT firms is the second most effective type, but the magnitude of which is very small. The remaining types of collaborative innovation have significant effects, although the magnitude of which is also small. *Firm-University* collaboration has been regarded as an important collaborative type in innovation research [69, 70], but the effect on technological convergence is negligible. Firm-GRI collaboration, which has served primarily as a policy instrument to supplement firms’ innovation resources, also has little effect on technological convergence.

## Discussions and conclusions

This paper examines the effect of collaborative innovation types on technological convergence in the context of the Korean ICT industry using panel data between 1980 and 2015. I find that inter-ICT firm collaboration has a positive and largest effect on technological convergence. This finding can serve as a basis to discuss the design of an effective incentive system. Below I elaborate the contributions and implications of this study.

This study contributes to the refinement of theories in innovation research. The results showed that the inter-ICT firm collaborative innovation has the largest effect on technological convergence, and the rest were statistically significant, but only minimally. The literature has suggested that collaborative innovation is a complex enterprise [71]. Without considering such complexity, understanding the effect of collaborative innovation can be misleading for policy-makers. For instance, firm-university collaboration has been widely believed to have positive externalities [69, 70]. The findings of this study show that firm-university collaborative innovation has only a negligible effect on technological convergence, at least in the Korean ICT industry.

This study can contribute to the development of theoretical foundations of the relationship between collaboration innovation and technological convergence. This study not only strengthens the existing claim that firms’ collaboration efforts can influence open innovation strategy to promote technological convergence but also identifies the most effective collaboration type for technological convergence. Since the empirical analysis of technological convergence is limited [29], the results can contribute to the expansion of discussions by strengthening existing arguments that collaboration innovation affects technological convergence [16, 33]. The existence of simultaneity also implies that the relationship between collaboration innovation and technological convergence is not one-sided, and the reverse causality needs to be considered. Finally, this study suggests that only a few types of collaboration innovations have a significant effect on technological convergence, suggesting that collaboration types can be considered as an essential factor in the theoretical discussion on the relationship between collaboration innovation and technological convergence.

Since ICT technology has a high possibility of convergence with other technology areas [33, 72], firms can use convergence as an innovation strategy to meet diversified technology needs. In this convergence environment, firms’ innovation strategies may face the resistance of existing path dependency [73]. Thus, to break path dependency, firms use collaborative innovation strategies such as open innovation [73]. However, despite the importance of collaboration, the connections between innovators can be incomplete. The government can regard this as a system failure and intervene in innovation activities [74]. A systematic approach to innovation claims that firms rely heavily on collaboration and interaction with other innovators [75]. An example of a system failure is the lack of connections or interactions among the organizations or institutions that constitute the innovation system [76]. Thus, governments can intervene to complement innovation systems and promote collaborative innovation. Specifically, governments can promote technological convergence by focusing subsidies on the types of collaboration that are most effective for technological convergence.

GRI has served as a government policy instrument for a long time and has played the role of supporting innovative resources mainly for firms that are unable to cover the innovation costs or lack the technology or workforce needed for innovation. Universities also play a role in supporting innovative resources for firms with insufficient innovation capacity, but they perform these activities mainly through incentives, unlike GRIs. Universities have relatively high autonomy in selecting innovation subjects when compared to GRIs. Although universities are not as sensitive to changes in technology demand as firms are, firms have a relatively high level of collaborative innovation aimed at producing convergent technology through a combination of heterogeneous technologies. Firms, especially large firms with high financial capacities, fund most research from their internal budgets. Additionally, firms are highly sensitive to changes in technology demand in the market for survival. Since the innovation process takes time, firms prefer to preempt the technology relatively quickly through collaborative innovation with other firms that have different technologies, rather than developing all the technologies as internal innovations.

Technological convergence has been believed to spur inter-firm interactions such as clustering and collaboration [77, 78]. Also, firms in the future IT industry were expected to use collaborative strategies for technological convergence [6]. However, there was a lack of evidence to support this claim. This study’s findings revealed a reverse causality in which technological convergence affects collaborative innovation in the case of inter-ICT firm collaborative innovation. The result of the endogeneity test implies that one ICT firm choosing another ICT firm as a collaborative innovation partner could be a strategic action of ICT firms for technological convergence. By confirming the existence of a reverse causality, this study can be a starting point for discussions about the strategic behavior of firms for technological convergence as well as the effect of technological convergence on collaborative innovation. The results indicate that open innovation strategies are not always useful for technological convergence. The effectiveness of firms’ open innovation strategies depends on the type of collaboration, and the effect of collaborative innovation with universities and GRIs on technological convergence is negligible. Therefore, before leveraging an open innovation strategy, firms should thoroughly investigate the type of partner and verify which partner best fits the goals they hope to achieve through collaborative innovation.

On the other hand, it is reasonable to argue that it needs to strengthen investment by collaboration type with relatively little effect on technological convergence, such as firm-university collaboration. While firms have a strong motivation to develop new technologies for commercialization through Schumpeterian competition [79, 80], universities are not exposed to this kind of competition. Universities have played a role in solving the market failure of innovation through development of basic technologies that are not developed in the market but have large external effects [81, 82]. However, because the integration between heterogeneous technologies based on ICT technology spread widely in recent years, universities need to focus on developing convergent technologies. The government could grant subsidies to strengthen universities’ capabilities in developing convergent technologies. This approach may be less effective in promoting technological convergence in the short term rather than strengthening subsidies for inter-firm collaboration but, in the long run, a university could contribute to its role in the convergence environment.

This study also has limitations. It focuses on determining which type of collaborative innovation produces the largest amount of convergent technology, and there is no discussion on the quality of convergent technology. If the quality of convergent technology produced by collaborative innovation differs, the government can implement the policy that gives the highest incentive to the collaborative innovation type that produces the highest-quality convergent technology. Therefore, subsequent studies should analyze which types of collaborative innovations produce the highest-quality convergent technology. Second, this study used joint patenting data to measure collaborative innovation due to limited data. Although patents have been widely used as proxies to measure innovation [34, 83], innovations that firms do not disclose cannot be measured by patenting activities. Subsequent studies are expected to verify whether similar results are obtained when using other measurements such as strategic alliances. Third, this study limited the unit of analysis to firms. The inclusion of GRI in the analysis may limit the use of existing control variables in regression analysis. Innovation mechanisms of firms and GRIs are different, and variables such as IPO and Conglomerate Affiliates are valid only in the firms’ innovation mechanism. Firms are the main drivers of innovation in the industry, but GRI also plays an important role in the innovation system. Subsequent studies are expected to include not only firms but also various types of innovators in the unit of analysis.

In conclusion, the importance of technological convergence has increased as the pattern of innovation competition has changed, centering on ICT-based technological convergence such as IoT technologies. Collaborative innovation is a crucial strategy to facilitate technological convergence. This study’s findings suggest that the effect of collaborative innovation may be sensitive to collaborative innovation types. A nuanced and subtle understanding of collaborative innovation and technological convergence can expand and refine the collective understanding and on-going conversations about innovation policy.

## Supporting information

S1 Table RENB regression. (DOCX)

S2 Table Hausman test (FENB vs. RENB). (DOCX)

S3 Table Poisson regression. (DOCX)

S4 Table Hausman test (IV Poisson GMM vs. Poisson). (DOCX)
