# Supplementary material for: The effect of collaborative innovation on ICT-based technological convergence: A patent-based analysis
Source: PLoS One. 2020 Feb 4;15(2):e0228616. doi: 10.1371/journal.pone.0228616 (PMC6999869; doi:10.1371/journal.pone.0228616)
Supplement: S1 Table — (DOCX) [file pone.0228616.s001.docx]

S1 Table. RENB regression

| Variable | Model (1) | Model (2) | Model (3) | Model (4) | Model (5) |
| --- | --- | --- | --- | --- | --- |
| **Independent Variables** | | | | | |
| Collab.Innov. | 0.0039***  (0.0004) |  |  |  |  |
| Firm-University |  | 0.0071***  (0.0010) |  |  |  |
| Firm-GRI |  |  | 0.0050***  (0.0010) |  |  |
| Inter-firm |  |  |  | 0.0227***  (0.0024) |  |
| Inter-ICT firm |  |  |  |  | 0.0596***  (0.0091) |
| **Control Variables** | | | | | |
| Ln(FirmSize) | 0.0518  (0.0563) | 0.0869  (0.0568) | 0.0414  (0.0574) | 0.0774  (0.0561) | 0.0658  (0.0561) |
| Ln(R&D Exp.) | 0.1668***  (0.0420) | 0.1549***  (0.0427) | 0.1708***  (0.0425) | 0.1510***  (0.0420) | 0.1596***  (0.0419) |
| Ln(Productivity) | -0.0090  (0.0772) | 0.0149  (0.0771) | -0.0162  (0.0775) | -0.0207  (0.0771) | -0.0154  (0.0764) |
| Conglomerate Affiliates | -0.3646*  (0.1755) | -0.3684*  (0.1753) | -0.3644*  (0.1758) | -0.3775*  (0.1756) | -0.3644*  (0.1754) |
| Firm Age | -0.0122*  (0.0061) | -0.0137*  (0.0061) | -0.0116  (0.0061) | -0.0155*  (0.0061) | -0.0138*  (0.0061) |
| Large Firms | 0.1361  (0.1515) | 0.1093  (0.1511) | 0.1416  (0.1514) | 0.1225  (0.1512) | 0.1340  (0.1513) |
| IPO | -0.4442***  (0.1211) | -0.4126***  (0.1210) | -0.4579***  (0.1225) | -0.5387***  (0.1223) | -0.4444***  (0.1205) |
| Year | 0.1202***  (0.0095) | 0.1170***  (0.0094) | 0.1212***  (0.0094) | 0.1205***  (0.0094) | 0.1194***  (0.0095) |
| **Constant** | -243.4704***  (18.8429) | -237.1636***  (18.7664) | -245.2931***  (18.7252) | -243.8032***  (18.7965) | -241.8219***  (18.8147) |
| **Observations** | 6,891 | 6,891 | 6,891 | 6,891 | 6,891 |
| **Log Likelihood** | -3733.8929 | -3739.8307 | -3742.957 | -3730.6348 | -3740.242 |
| **Ln(r)** | 0.2197  (0.1077) | 0.2181  (.0916) | 0.2112  (0.0915) | 0.2212  (0.0918) | 0.2166  (0.0916) |
| **Ln(s)** | -0.8683  (0.1083) | -0.8648  (0.1079) | -0.8749  (0.1075) | -0.8662  (0.1078) | -0.8695  (0.1077) |
| **LR Chi-bar-squared** | 1415.06*** | 1397.92*** | 1452.71*** | 1365.38*** | 1395.65*** |
| **Prob > Chi-bar-squared** | 0.000 | 0.000 | 0.000 | 0.000 | 0.000 |

Note: * p<0.05; ** p<0.01; *** p<0.001; Standard errors in parentheses.
