# Supplementary material for: The effect of collaborative innovation on ICT-based technological convergence: A patent-based analysis
Source: PLoS One. 2020 Feb 4;15(2):e0228616. doi: 10.1371/journal.pone.0228616 (PMC6999869; doi:10.1371/journal.pone.0228616)
Supplement: S2 Table — (DOCX) [file pone.0228616.s002.docx]

S2 Table. Hausman test (FENB vs. RENB)

|  | Model (1) | Model (2) | Model (3) | Model (4) | Model (5) |
| --- | --- | --- | --- | --- | --- |
| Chi-Squared | 66.39*** | 74.74*** | 107.35*** | 73.83*** | 43.89*** |
| Prob > Chi-Squared | 0.0000 | 0.0000 | 0.0000 | 0.0000 | 0.0000 |
| Regression Model | FENB | FENB | FENB | FENB | FENB |

Note: * p<0.05; ** p<0.01; *** p<0.001
