# Supplementary material for: The effect of collaborative innovation on ICT-based technological convergence: A patent-based analysis
Source: PLoS One. 2020 Feb 4;15(2):e0228616. doi: 10.1371/journal.pone.0228616 (PMC6999869; doi:10.1371/journal.pone.0228616)
Supplement: S3 Table — (DOCX) [file pone.0228616.s003.docx]

S3 Table. Poisson regression

| Variable | Model (1) | Model (2) | Model (3) | Model (4) a | Model (5) |
| --- | --- | --- | --- | --- | --- |
| **Independent Variables** |  |  |  |  |  |
| Collab.Innov. | 0.0017*** (0.0001) |  |  |  |  |
| Firm-University |  | 0.0040***  (0.0002) |  |  |  |
| Firm-GRI |  |  | -0.0002  (0.0003) |  |  |
| Inter-firm |  |  |  | 0.0188***  (0.0006) |  |
| Inter-ICT firm |  |  |  |  | 0.0420***  (0.0015) |
| **Control Variables** |  |  |  |  |  |
| Ln(FirmSize) | 0.4500***  (0.0160) | 0.4737***  (0.0160) | 0.4793***  (0.0162) | (Omitted) | 0.4706***  (0.0160) |
| Ln(R&D Exp.) | 0.4190***  (0.0122) | 0.3977 ***  (0.0123) | 0.4393***  (0.0122) | 0.6087***  (0.0062) | 0.3889***  (0.0122) |
| Ln(Productivity) | -0.2594***  (0.0228) | -0.2358***  (0.0228) | -0.2557***  (0.0225) | -0.4207***  (0.0228) | -0.2215***  (0.0227) |
| Conglomerate Affiliates | 0.0397  (0.0586) | 0.0402  (0.0586) | 0.0241  (0.0587) | 0.1379*  (0.0582) | 0.0364  (0.0586) |
| Firm Age | -0.0292***  (0.0013) | -0.0311***  (0.0014) | -0.0301***  (0.0013) | (Omitted) | -0.0317***  (0.0014) |
| Large Firms | 0.5570***  (0.0433) | 0.5648***  (0.0433) | 0.4728***  (0.0435) | 0.8859***  (0.0408) | 0.5769***  (0.0432) |
| IPO | -1.0161***  (0.0571) | -0.8966***  (0.0563) | -0.9005***  (0.0577) | -0.8840***  (0.0567) | -0.8859***  (0.0563) |
| Year | 0.0731***  (0.0026) | 0.0712***  (0.0026) | 0.0675 ***  (0.0026) | 0.0495***  (0.0026) | 0.0734***  (0.0027) |
| **Constant** | -151.6265***  (5.2714) | -147.8649***  (5.2471) | -140.7343***  (5.1004) | -103.0592***  (5.1228) | -152.2376***  (5.3034) |
| **Observations** | 6,891 | 6,891 | 6,891 | 6,891 | 6,891 |

Note: * p<0.05; ** p<0.01; *** p<0.001; Standard errors in parentheses.

a Model (4) is estimated to exclude the *Ln(FirmSize)* and *Firm Age* variables from the existing model so that it is the same as the IV Poisson GMM model to perform the Hausman test.
