# Supplementary material for: The effect of collaborative innovation on ICT-based technological convergence: A patent-based analysis
Source: PLoS One. 2020 Feb 4;15(2):e0228616. doi: 10.1371/journal.pone.0228616 (PMC6999869; doi:10.1371/journal.pone.0228616)
Supplement: S4 Table — (DOCX) [file pone.0228616.s004.docx]

S4 Table. Hausman test (IV Poisson GMM vs. Poisson)

|  | Model (1) | Model (2) | Model (3) | Model (4) | Model (5) |
| --- | --- | --- | --- | --- | --- |
| Chi-Squared | 0.75 | 2.52 | 0.65 | 1.97 | 22.68** |
| Prob > Chi-Squared | 0.9998 | 0.9802 | 0.9999 | 0.9613 | 0.0069 |
| Regression Model | Poisson | Poisson | Poisson | Poisson | IV Poisson GMM |

Note: * p<0.05; ** p<0.01; *** p<0.001
